# Traditional Dietary Patterns and Risk of Mortality in a Longitudinal Cohort of the Salus in Apulia Study

**DOI:** 10.3390/nu12041070

**Published:** 2020-04-12

**Authors:** Roberta Zupo, Rodolfo Sardone, Rossella Donghia, Fabio Castellana, Luisa Lampignano, Ilaria Bortone, Giovanni Misciagna, Giovanni De Pergola, Francesco Panza, Madia Lozupone, Andrea Passantino, Nicola Veronese, Vito Guerra, Heiner Boeing, Gianluigi Giannelli

**Affiliations:** 1 Population Health Unit —“Salus in Apulia Study”—National Institute of Gastroenterology “Saverio de Bellis”, Research Hospital, Castellana Grotte, 70013 Bari, Italy; zuporoberta@gmail.com (R.Z.); castellanafabio@hotmail.it (F.C.); luisalampignano@gmail.com (L.L.); ilariabortone@gmail.com (I.B.); gmisciagn@libero.it (G.M.); f_panza@hotmail.com (F.P.); madia.lozupone@gmail.com (M.L.); 2 Data Analysis Unit, National Institute of Gastroenterology “Saverio de Bellis”, Research Hospital, Castellana Grotte, 70013 Bari, Italy; rossydonghia@gmail.com (R.D.); vito.guerra@irccsdebellis.it (V.G.); boeing@dife.de (H.B.); 3 Clinical Nutrition Unit, Medical Oncology, Department of Biomedical Science and Human Oncology, University of Bari, School of Medicine, Policlinico, Piazza Giulio Cesare 11, 70124 Bari, Italy; gdepergola@libero.it; 4 Neurodegenerative Disease Unit, Department of Basic Medicine, Neuroscience, and Sense Organs, University of Bari Aldo Moro, 70121 Bari, Italy; 5 Department of Cardiology and Cardiac Rehabilitation, Scientific Clinical Institutes Maugeri, IRCCS Institute of Cassano Murge, 70020 Bari, Italy; andrea.passantino@icsmaugeri.it; 6 Azienda ULSS 3 Serenissima, Primary Care Department, District 3, 30174 Venice, Italy; ilmannato@gmail.com; 7 German Institute of Human Nutrition Potsdam-Rehbrücke, 14558 Nuthetal, Germany; 8 Scientific direction, National Institute of Gastroenterology “Saverio de Bellis”, Research Hospital, Castellana Grotte, 70013 Bari, Italy; gianluigi.giannelli@irccsdebellis.it

**Keywords:** healthy diet indexes, food intake, apulia, mind index, dash index, med-diet score

## Abstract

There is still room for further studies analyzing the long-term health impact of specific dietary patterns observable in regions belonging to the Mediterranean area. The aim of the study is to evaluate how much a diet practiced in southern Italy is associated to a risk of mortality. The study population included 2472 participants first investigated in 1985, inquiring about their frequencies of intake of 29 foods using a self-administered questionnaire covering the previous year. The population was followed up for mortality until 31 December 2017. Cox-based risk modeling referred to single foods, food groups, the results of principal component analysis (PCA), and a priori indexes. Single food analysis revealed eggs, fatty meat, and fatty/baked ham to be inversely associated with mortality. Furthermore, one of the 5 PCA derived dietary patterns, the “Farmhouse” pattern, showed a higher hazard ratio (HR), mostly driven by dairy products. In subsequent analyses, the increased risk of mortality for fresh cheese and decreased risk for fatty ham and eggs were confirmed. The a priori diet indexes (Italian Meddiet, Meddietscore, Dietary Approaches to Stop Hypertension (DASH), and Mediterranean–DASH Intervention for Neurodegenerative Delay diet (MIND) indexes) showed borderline inverse relationships. In a Mediterranean population with an overall healthy diet, foods such as eggs and fatty meat, reflecting dietary energy and wealth, played a role in prolonging the life of individuals. Our study confirms that some dairy products might have a detrimental role in mortality in the Mediterranean setting.

## 1. Introduction

In the last decades, life expectancy has increased in most parts of the world and population aging is now a global phenomenon, especially in developed countries. In Europe, the life expectancy of the Italian population is the second highest, being two years longer than the European average, based on a gain of 2.8 years in life expectancy between 2000 to 2015. However, this advantage of the Italian population in terms of life expectancy compared to their other European peers is not reflected in a similar gain in disability-free years, as reported by the Institute for Health Metrics and Evaluation (IHME).

This relatively privileged situation in terms of life expectancy in Italy might be related to the lifestyle, in particular, the diet consumed in this country. Meta-analyses with mortality as endpoint identified a number of food groups making up the Italian diet that are associated with a reduced risk of mortality, such as high intake of vegetables, fruits, fish, legumes, nuts, and whole grains, and a low intake of red and processed meat [1,2,3,4]. Accordingly, dietary indexes defined a priori, including those foods such as the Mediterranean-Diet score [5], the Healthy Eating index [6], the Alternate Healthy Eating index [7], or the Dietary Approaches to Stop Hypertension index (DASH) [8], were found to be associated with a reduced risk of all-cause mortality.

The dietary pattern in Italy is the heritage of millennia of exchanges of people, cultures, and foods among all countries around the Mediterranean basin, ever since the Greek and Roman expansions. This diet is rich in plant foods (cereals, fruit, vegetables, nuts, and legumes), with olive oil as the principal source of added fat, combined with a moderate intake of animal foods, including dairy products, and drinking wine especially during meals [9,10].

Fortunately, there are areas in Italy where the traditional Italian diet is still practiced by a large part of the population. Recently, we confirmed that the local population of Castellana Grotte, located in Apulia, has shown a very stable dietary behavior over the past years (Castellana et al. 2019), despite some changes in the last decades [11]. In this town and other parts of Italy, most of the foods are produced locally, such as fruits, vegetables, and legumes. In addition, the Apulian diet includes specific regional varieties such as orecchiette as the main pasta, and the bread is produced with semolina flour, an unrefined type of flour with a rich dietary fiber content (Castellana et al. 2019).

The long tradition of research into diet, launched by the National Institute of Gastroenterology “S. De Bellis” (IRCCS) with the establishment of a prospective cohort study in 1985, involving more than 2500 participants from Castellana Grotte, a small town south of Bari and the seat of the Institute, allowed investigation of the long-term role of food intake for risk of mortality to be investigated in a Mediterranean diet setting. The risk modeling included the investigation of single foods, food groups, the results of principal component analysis (PCA), and *a priori* healthy diet indexes.

## 2. Materials and Methods

### 2.1. Study Population:

In the beginning of the 1980s, the IRCCS De Bellis participated in the Multicenter Italian study on Cholelithiasis (MICOL) [12] with the aim of prospectively investigating the role of lifestyle and nutrition in gastrointestinal and other chronic diseases, including cholelithiasis [13,14]. In 1985, a random sample of 3500 subjects (2000 men and 1500 women) aged ≥30 years was drawn from the electoral roll of Castellana Grotte (17,334 residents at the 1981 census); 2472 (1429 men and 1043 women) agreed to participate (70.6% response rate). Thus, the study population could be considered as a representative sample of the population of Puglia of that time (1980s). The cohort was reexamined several times over the past three decades (Castellana et al. 2019). In the current investigation, we took into account only the first survey in 1985 (M1) with a follow-up on mortality until 31 December 2017. The mortality data were obtained from the Electronic Health Records of the Regione Puglia. All participants agreed to take part in the MICOL study, giving informed consent and allowing sharing of their medical data, including the death events and causes of death, with the study team. All study procedures were in accordance with the ethical standards of the institutional research committee and with the 1964 Helsinki declaration. Every single examination and informed consent form was approved by the Institutional Review Board of the National Institute of Gastroenterology and Research Hospital. All study information is stored in electronic databases that are protected according to Italian privacy laws.

### 2.2. Dietary and Lifestyle Variables

A self-administered ad hoc questionnaire was provided by the MICOL Group and assessed dietary habits of the previous year. In the questionnaire, the intakes of 29 foods were inquired about as frequencies per week in categories from 0 to 4 (0 = never, 1= rarely, 2 = occasionally, 3 = frequently, 4 = daily). Separately from foods, the use of olive oil was assessed in three categories (0 = never, 1 = occasionally, 2 = frequently) and the consumption of wine in six categories ranging from 0 consumption to more than 2 L a day. The self-administered questionnaire was checked for completeness by a physician during an interview at the study center. The interview was conducted by a medical doctor and included questions on lifestyle aspects such as educational level, physical activity, and smoking. Additionally, at the interview, anthropometric data were obtained, such as weight (kg) and height (cm), measured with SECA 700 and SECA 220 (Seca GmBH and Co., Hamburg, Germany), from which the body mass index (BMI) was derived, calculated as the ratio of weight (kg) to height squared (m^2^). Multimorbidity status was defined as the co-presence of two or more chronic diseases measured by the presence of the following conditions: diabetes, hypertension, peptic ulcer, cholangiolithiasis, myocardial infarction, hepatic cirrhosis or other liver diseases, inflammatory bowel diseases, major infectious diseases, leukemia or other blood chronic diseases, viral hepatitis, AIDS [15]. All diseases were assessed using a general medical history questionnaire administered by an expert physician.

### 2.3. Food Group and Dietary Patterns

#### 2.3.1. Food Groups

Food groups were formed by summarizing the frequencies of single foods such as legumes (peas, beans, lentils, fava beans, chickpeas), vegetables (raw and cooked vegetables), fresh cheese (sheep and cow ricotta, fiordilatte, mozzarella, smoked provola, and other cheese), red meat (low-fat meat and fatty meat), processed meat (cotechino, zampone, cured meat, sausages, lean ham, fatty ham), fish and seafood (e.g., mussels, tuna, oysters, salmon), and sweets (croissant, buns, and chocolate).

#### 2.3.2. A Priori Dietary Pattern

Four healthy/preventive diet indexes were calculated according to published algorithms that were adapted to the available food information: Meddiet score [16], DASH-diet index [8], MIND-diet index [17], and Italian Meddiet index [18]. Each index was created by defining the dietary components that contributed to the index, adding the frequencies of the foods contributing to the components, and using the median value of the total sample as the cut-off, if not otherwise instructed.

#### 2.3.3. A Posteriori Dietary Patterns

A robust algorithm of data reduction, the PCA [19], was applied to the 29 food frequencies to summarize new variables, such as principal components, that we defined as new dietary patterns. We selected the first five principal components according to the “percentage of variance” criterion; this means that the cumulative percentage (eigenvalue x 100) of variance explained from the components 1 to 5 covered 95% of the information. Subsequently, two trained nutritionists (L.L. and F.C.) interpreted the PCA components and agreed on a proper naming of the pattern. The naming was approved by a third senior nutritionist (R.Z.).

#### 2.3.4. Predictive Variables for the Outcomes

In order to select the most predictive foods for mortality, we used a machine-learning algorithm, the random survival forest (RSF) [20]. This algorithm was able to select the best predictor in a set of 29 variables. The latter were filtered using variable importance criterion (VIMP): large importance values indicated variables with higher predictive power. Furthermore, to better improve the data output interpretation of RSF, we implemented an automated backward elimination procedure; this represents a corroborated method for highly correlated complex data, to avoid noise in data outputs [21].

#### 2.3.5. Mortality Assessment

In order to explore the association of dietary patterns and foods on cause-specific mortality, we categorized findings on the basis of three different causes. Firstly, death for all-causes, secondly deaths subsequent to all types of cancers, finally, deaths due to cardiovascular and cerebrovascular diseases (mostly strokes and myocardial infarction). All causes were taken from the Apulia Region Public Health Records, assessed using International Classification of Diseases (ICD)-10 classification. We limited the investigation to the most common causes of death because they provide enough cases to run the Cox proportion models.

### 2.4. Statistical Analysis

Data are reported as means ± standard deviations (M ± SD) for continuous measures, and frequency and percentages (%) for all categorical variables. We used Cox modeling to explore the relationship between overall and specific-cause mortality and explanatory variables, estimating the hazard rate ratio (HR) and 95% confidence interval (95% CI). The HR was taken as an approximation of the relative risk (RR). Each subject was censored by the end of follow-up (31 December 2017), the date of death or the date of termination of study participation. For Cox modeling of the food variables, the following covariates as potential risk factors, were used: age (continuous variable), sex (dichotomous variable), BMI (continuous variable), educational level (categorized as no schooling, primary school, secondary school/university degree), smoking (categorized as never-smoker and former smoker/current smoker (≤20 cigarettes/d and >20 cigarettes/d), multimorbidity (two categories: >2 and <2 prevalent diseases), wine (categorized as non-consumer and consumer of less than 1 L and more than 1 L a day), and olive oil (categorized as frequent users and less frequent users).

Various statistical approaches were used to identify the food groups associated with mortality. Firstly, the 29 food groups were related to mortality, adjusted for covariates (see above). Significant results according to the 95% CI are highlighted in bold in the table. These food groups were further used in a multiple Cox regression model applying the backward stepwise method. Only variables that showed associations with *p* < 0.10 were left in the models. The foods selected in such a way were related to mortality, mutually adjusted, and the variables that showed associations with *p* < 0.10 were left in the models. In further approaches, it was investigated whether food groups related to a risk of mortality, in the initial analysis with covariates, still dominated the results regarding the outcome mortality. One approach was to form broader food groups and relate these groups to mortality. Other approaches were of a more exploratory nature. The a priori (Meddiet score, DASH, and MIND index) and a posteriori (PCA) food patterns were related to a risk of mortality. Furthermore, the foods most predictive of the RSF were also related to the outcome. Finally, we related the foods associated with mortality also to cause-specific mortality. All analyses were performed using StataCorp 2017 Stata (Release 15 statistical software, College Station, TX, StataCorp LLC).

## 3. Results

Baseline characteristics of the M1 cohort population are presented in Table 1. These variables were also selected as confounders for the risk modeling of the dietary variables and hence, also yielded the mutually adjusted hazard ratio (HR). The M1 population was nearly balanced regarding gender, with a slight dominance of men, was middle-aged, being about 50 years old on average, most had low education, featuring 5 years of schooling or less, and obese according to the traditional classification of BMI. Smoking was not widespread for the time period of the mid-1980s to 1990s, compared to other populations, and was more common among men (45.7% men and 12.8% women). Drinking more than one liter per day of wine showed a clear dominance among men compared to women (19.1% against 1.5%). Olive oil dominated as a fat source and one-third declared frequent use.

Of the 2472 participants, 990 died. This equals 400 deaths per one million person years. Cox regression modeling revealed a reduced risk of mortality for females and for a frequent use of olive oil. Increased risks were found for age, smoking, and body mass index. All estimates were in line with current knowledge about lifestyle factors that influence the risk of mortality.

The frequency information covered 29 food items. The mean frequencies—expressed as consumption per day—are shown for the total group and for men and women (Table 2). The frequencies of semolina type bread and fruit stood out, showing a frequency of several times per day. Raw and cooked vegetables were eaten every second day, alternating with legumes. Consumption of milk and dairy products such as ricotta or mozzarella was widespread (Table 2). Intake of men and women did not differ in principle in terms of frequency, allowing a combined analysis across gender. The main difference between gender regarded semolina type bread eaten by men compared to women (+0.07 per day), followed by desserts (−0.04), fatty meat (+0.04), skimmed milk (−0.04), sheep ricotta (−0.04), cow ricotta (−0.04), and raw and cooked vegetables (−0.04).

**Figure 1:**
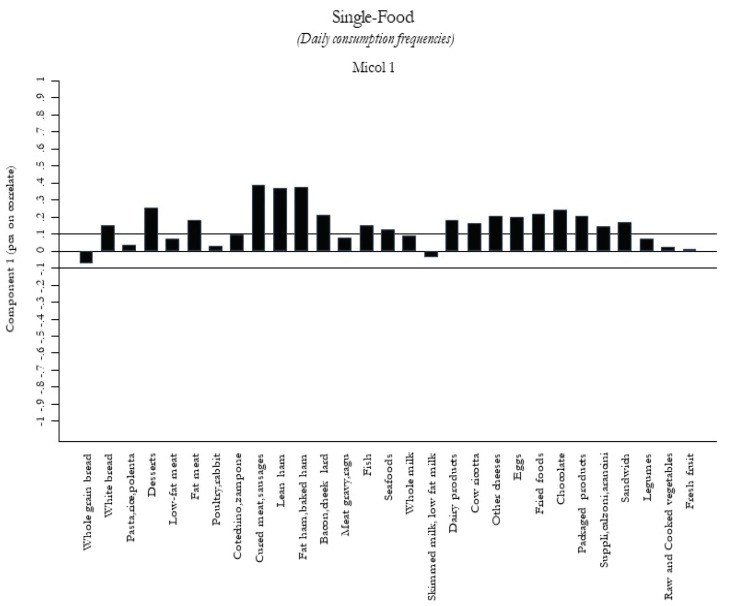
First dietary pattern, “Energy-Rich Foods Pattern”, derived with principal component analysis.

**Figure 2:**
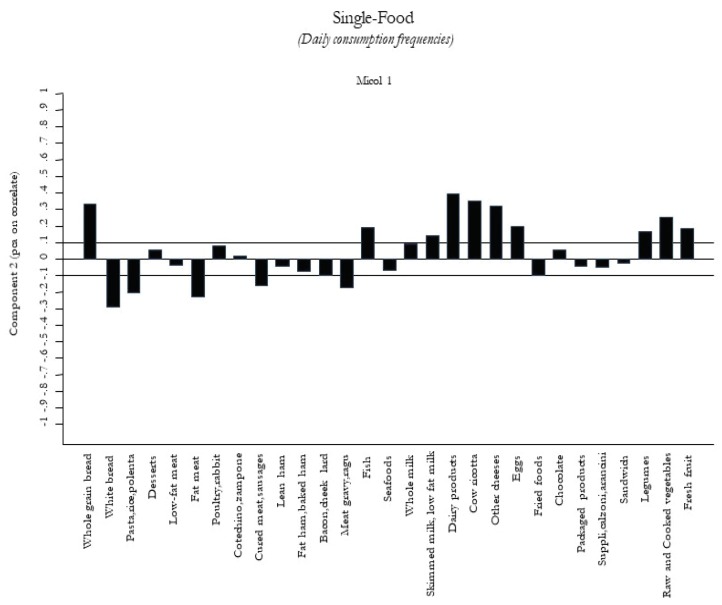
Second dietary pattern “Farmhouse Diet” derived with principal component analysis.

**Figure 3:**
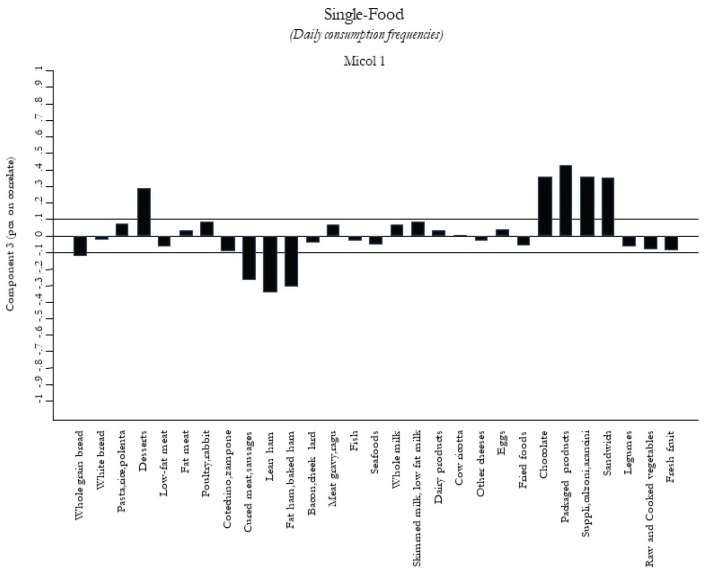
Third dietary pattern, “Sweets Pattern”, derived with principal component analysis.

**Figure 4:**
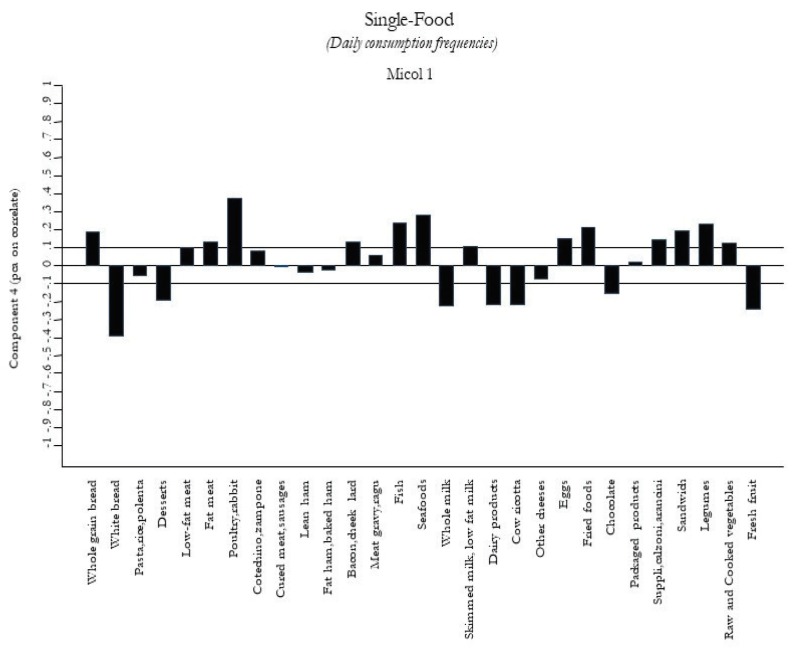
Fourth dietary pattern, “Winter Pattern”, derived with principal component analysis.

**Figure 5:**
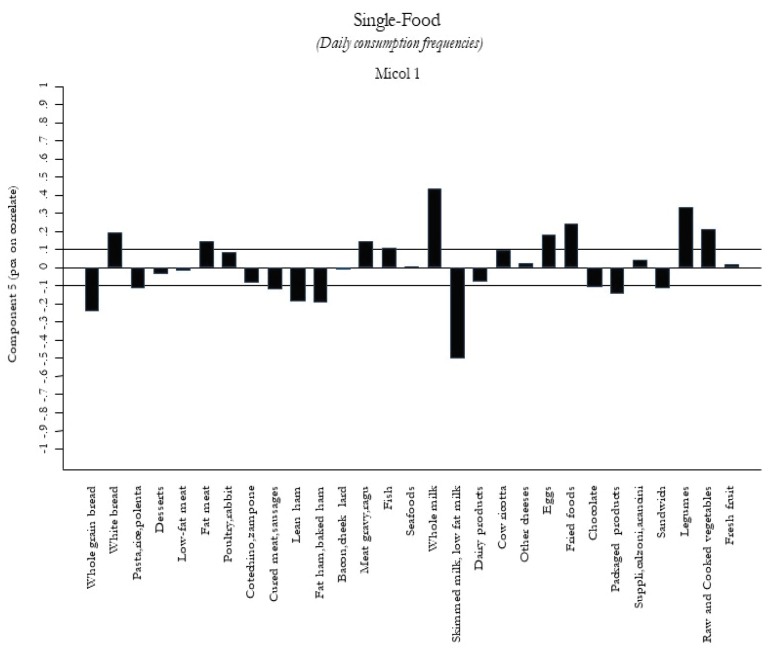
Fifth dietary pattern, “Elderly Pattern”, derived with principal component analysis

According to the percentage of variance, we selected the first five components as food patterns consumed by the Castellana population. The 5 food patterns are shown in Figure 1 Figure 2 Figure 3 Figure 4 Figure 5. The first food pattern (Figure 1) reflected high frequencies, particularly of cured meat, sausages, lean ham, and bacon, followed by desserts, chocolate, and packaged/fried foods. It was named the “Energy-Rich Foods Pattern”. The second food pattern (Figure 2) reflected a high-frequency intake of all dairy products, including fresh and other cheeses, integrated in a diet with a lot of vegetables, legumes, fruits, and semolina type bread. It was named the “Farmhouse Diet”. The third food pattern (Figure 3) reflected a high frequency of intake of sweets such as desserts, chocolate, and package products. It was named the “Sweets Pattern”. The fourth food pattern (Figure 4) reflected a high frequency of intake of whole grains, poultry, fish, seafood, and legumes. It was named the “Winter Pattern”. The fifth food pattern (Figure 5) reflected a high consumption of whole milk, semolina type bread, legumes, and vegetables. It was named the “Elderly Pattern”.

In Table 3, the hazard ratios (HR) for mortality for intake of food per week are shown. Only a few food items were significantly related to risk; this did not include the obvious candidates such as fruit and vegetables. Instead, fatty meat, fatty/baked ham, and eggs showed an inverse relationship between frequency and risk of mortality. Further suggestive but non-significant relations were seen for semolina type bread (inverse), and sheep ricotta, mozzarella, smoked provola (positive).

In the first lines of Table 4, the HRs for the combined frequencies reflecting food groups are shown. This analysis now showed a significantly increased risk with increased frequency of ricotta type cheese, and some but non-significant suggestions that processed meat in general was associated with a reduced risk. The previous risk analyses of foods did not include the adjustment for other foods, which is in line with the exploratory nature of the study. The role of such adjustments was investigated via backward selection, starting from a fully adjusted model. The result with the first five foods is shown in Table 4. It appeared that only two (fatty/baked ham, eggs) of the three in the unadjusted risk models for other foods identified foods (fat meat, fatty/baked ham, eggs) were still related to risk but sheep ricotta, previously suggestively related to risk, now reached significance. The fifth food analyzed with the backward approach did not reach significance at the 5% level.

**Figure 6:**
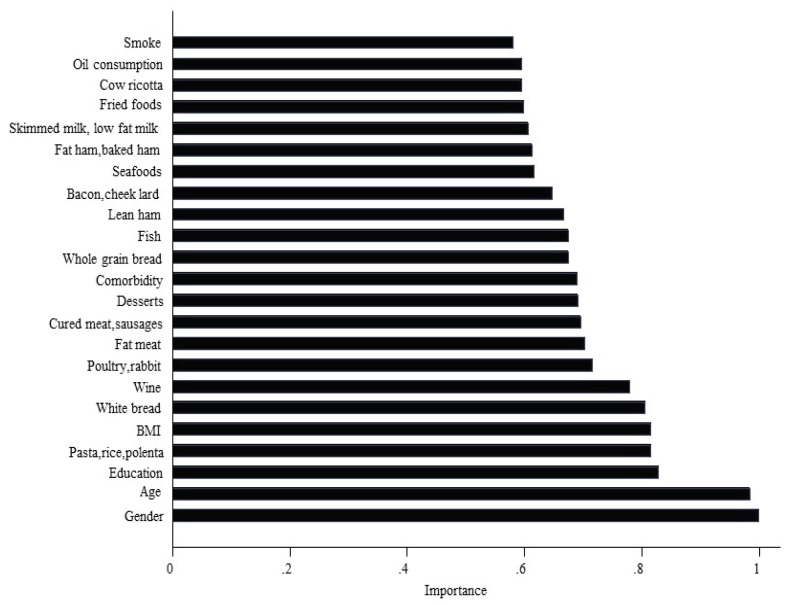
RSF, Importance score of predictor variables with covariates on all foods and olive oil with covariates on all foods and olive oil.

The role of dietary variables was also investigated by random survival forest (RSF), a machine learning algorithm ranking the prediction power through a backward selection. Starting with the most important variable to subdivide the sample, the other variables were ranked according to the importance (prediction power) criteria (Figure 6). Figure 6 clearly shows that dietary variables play an important role, nowithstanding the adjustment for all confounders. The 5 most important food variables ranked by RSF were again used in the traditional Cox regression (Table 4) mutually adjusted in the same model. The selection procedure by RSF identified a different set of variables compared to the regression backward selection. Interestingly, the RSF set included fatty meat, which was again significantly inversely related to risk despite different adjustments, as before.

The relations of food patterns to risks of mortality are also shown in Table 4. Only the “Farmhouse Pattern” was significantly related to an increased risk of mortality. A decreased risk was associated with increasing scores for the Energy-Rich Food Pattern and the Winter Pattern, but this did not reach significance. The other two food patterns did not show any suggestive relationship to the risk of mortality.

Additionally, we investigated whether a priori healthy diet indexes such as the Meddiet score, Italian MedDiet Index, and the DASH and MIND indices were related to risk, created with the sum of the frequencies for each food included in the scores [8,17,22]. No significant association was found for the DASH index (HR 1.01, 95% CI 0.97 to 1.10). However, the MedDiet score, Italian MedDiet index, and the MIND index showed borderline inverse associations with risk of mortality (HR 0.98, 95% CI 0.97 to 1.00; HR 0.95 95% CI 0.90 to 1.00; HR 0.96, 95% CI 0.92 to 1.00, respectively).

In Table 5, we show the cause-specific results for the variables with significant associations with general mortality (Table 5). Not all of the variables showing associations with general mortality could be linked to the two causes of death, such as the “Farmhouse Diet” and fatty meat, fatty ham, and fresh cheese. However, we observed that eggs were inversely associated with risk of CVD death (HR 0.74, 95% CI 0.55 to 0.99) but not with cancer death (HR 0.85, 95% CI 0.65 to 1.10) (Table 5).

## 4. Discussion

This prospective investigation carried out in a population-based cohort of 2472 Italian middle-aged participants of Castellana Grotte (Puglia Region, Italy) addressed dietary and other lifestyle factors and risk of mortality over a period of more than three decades. It appeared that traditional Mediterranean dietary variables such as fruit, vegetables, and fish were not associated with reduced individual mortality in this study population, but other dietary variables such as fatty meat and eggs were. The study also identified the “Farmhouse” pattern as positively associated with the risk of mortality. This pattern included, in particular, fresh cheese products that showed to be equally on the risky side of the analyses with food and food groups.

The study population of Castellana followed a traditional rural Mediterranean diet with a high frequency of consumption of fruit, vegetables alternating with legumes, a bread mostly based on a semolina type of meal, and local pasta. Whole bread was uncommon at the time of examination [11]. Most of the study population had attended school only for a few years and worked in the agricultural sector or in small enterprises. This might have led to a more uniform diet in terms of the major components. Other than this, traditional socioeconomic and lifestyle factors such as gender, low education, high BMI, and smoking showed an increased risk of mortality, even when mutually adjusted, confirming the internal validity of the study.

The Mediterranean lifestyle includes the consumption of wine, mostly at meals (nearly 11% of the population drank more than one liter per day), and olive oil as the traditional fat source (nearly 35% reported frequent use). Both types of lifestyle habits were taken into consideration in all dietary analyses as adjusting factors. Whereas heavy wine consumption did not increase the risk of mortality, probably due to lack of a detailed quantification of daily intake, the frequent use of olive oil showed a reduced association with risk compared to moderate and less use.

Olive oil has a remarkably strong effect on health, especially extra virgin olive oil (EVOO). EVOO is the dominant type of oil consumed in the rural area of Apulia, and is well-known to be related to a decreased risk of chronic diseases such as cardiovascular disease (CVD) and to cardiometabolic risk factors (inflammation, oxidative stress, coagulation, platelet aggregation, endothelial dysfunction, obesity, and type 2 diabetes mellitus) [23,24]. Dietary patterns, including olive oils, had been linked to a reduced risk of mortality in the Spanish and the Italian arms of the EPIC study [25]. The phenolic compounds of EVOO, such as hydroxytyrosol and oleuropein, are considered as the driving forces of the health implications of its use, and to a lesser extent, the composition of the fatty acids [26].

Despite the fact that none of the other food patterns was related to the risk of mortality, it is interesting to note that study participants with a diet more closely related to the “Energy-Rich Food” or the “Winter” patterns showed a tendency to a lower risk of mortality. These two patterns shared a high frequency of consumption of energy and protein-rich foods such as meat, white and red meat, chocolate, desserts, fish, seafood, legumes, and eggs, and had the semolina type of bread as a further source of energy in the form of carbohydrates. In this rural setting, the availability of dietary energy seemed to be a factor of individual survival. In line with these thoughts, the results of the food analyses identified fatty meats and eggs as associated with a lower risk of mortality. We do not claim a direct link of fatty meat with the risk of mortality but consider dietary energy as a potential indirect link. This type of food could also be linked with a fairly good economic situation. The finding regarding eggs appeared to be in accordance with the latest study results in the Mediterranean population. In view of the dietary cholesterol debate, it has been overlooked that eggs are a source of high-quality protein, vitamins of the B-complex, folate, fat-soluble vitamins, and several essential minerals. The EPIC study in Spain was the first that investigated egg consumption in a large free-living Mediterranean population. Despite their failure to find a relation with overall or specific cause mortality, they found an inverse relation with death due to nervous system alterations, predominantly Alzheimer’s and Parkinson’s diseases [27]. However, epidemiological data about the association between egg consumption and overall mortality is still limited, despite some reports of no link [28,29], particularly in Mediterranean populations.

Regarding specific causes of death, our results showed that a high frequency of consumption of eggs is associated with a reduced risk of death due to CVDs and stroke. In line with this finding, it is suggested that eggs may even promote some heart-healthy effects based on a study in which HDL cholesterol increased with the consumption of eggs during a moderate carbohydrate restriction in overweight individuals [30], Otherwise, our finding regarding egg consumption is not corroborated in other studies and meta-analyses regarding stroke [29,31].

Milk and dairy products were part of the “Farmhouse” pattern and constitute an important source of energy as well as macro- and micronutrients in most Western countries; however, intakes differ largely between populations [32]. Study results regarding the long-term effects of those foods on health showed contradictory results [33,34]. This might be due to the fact that some studies were focused on the fat content of dairy products [35] and others on the impact of fermented and non-fermented dairy products [36,37]. Our results show that the consumption of non-fermented dairy products like sheep and cow ricotta, mozzarella, and smoked provola increased the risk of mortality, even after adjustment for other dietary factors. Similar results were reported by two other studies [38,39] conducted over the same time period in the Mediterranean area. Both investigations explored dietary habits in study populations in Greece. In this context, we should take the production process of such dairies, which requires salt, into account. In the late 1980s, the custom of making ricotta and cheese at home using milk local farming was still widespread in Puglia. A generous amount of salt was added to this dairy product [40]. Even in the current food composition tables prepared by the National Institute of Nutrition of Rome [41], 100 g of mozzarella contains around 200 mg of sodium (equal to 1.5 g of salt for a portion of 300 g) and smoked provola around 300 mg. We suppose that at the time of our data collection, it was common to use a much higher amount of salt in order to increase the palatability and shelf life of the products.

Advantages of this study include its long-term prospective observation (34-year follow up), the sample size, with a sufficient number of participants to address the research question, and the generalizability of the results to the south Italian population, the use of a larger number of foods to assess dietary habits, and the use of different statistical approaches.

The most important study limitation is the use of frequencies of foods instead of calculating quantitative daily intake. This type of measure could increase the bias that is usually associated with a retrospective dietary assessment over a period of a year, as compared to true intake, and also increase the bias that might be related to the risk estimates. To note, the survey questionnaire used in this study was not tested for its reliability and validity, and our results could have also been influenced by the subsequent changes in participants’ dietary habits over the follow-up years since we used information from the first survey only. The assessment method could also be affected by lifestyle variables and socioeconomic status. In order to compensate for these influences, we always adjusted all dietary models for other lifestyle factors (sex, age, BMI, education, smoking, comorbidities, wine, and olive oil consumption). Sensitivity analyses were used in addition to considering confounding by other foods. Also, we limited the investigation to the most common causes of death. We cannot rule out the possibility of residual confounding by factors that have not been evaluated or are suboptimally measured. One of these factors could be the changing dietary habits, which partly occurred in this study population over time periods [11].

In conclusion, in this area of a prevalent Mediterranean diet, with high consumption of vegetables, legumes, and fruits, other dietary factors are driving individual survival. However, olive oil still appears to be a dominant food factor for decreased mortality. The traditional Mediterranean diet, together with high-energy foods, could explain the higher life expectancy of the Italian population according to our analysis. The issue of dairy foods remains controversial and is a topic warranting further investigation.

## Figures and Tables

**Table 1:** Baseline characteristics of MICOL 1 subjects (*n* = 2472) and associated hazard ratios, adjusted for age and sex, and mutually adjusted.

Variables	Frequencies *	HR of Mortality Mutually Adjusted (95% CI)
Gender (Female) (%)	1043 (42.19)	0.65 (0.56 to 0.75)
Age (yrs) (M±SD)	48.00 ± 10.71	1.12 (1.11 to 1.13)
Education (%)		
Low (≤ 5years)	1657 (67.03)	1
Medium (8 years)	457 (18.49)	0.95 (0.79 to 1.16)
High (> 8years)	358 (14.48)	0.89 (0.70 to 1.13)
Smoking (%)	788 (31.88)	1.63 (1.40 to 1.89)
Body Mass Index (kg/m²)	27.48±4.55	1.04 (1.02 to 1.05)
Wine (≥ 1 lt/day) (%)	289 (11.69)	0.96 (0.79 to 1.16)
Comorbidity (≥ 2) (%)	154 (6.23)	1.20 (0.97 to 1.49)
Frequent use of olive oil (%)	864 (34.95)	0.84 (0.73 to 0.96)

* Proportion for categorical and means and standard deviation for continuous variables.

**Table 2:** Foods from the questionnaire, expressed as frequency per day.

Foods	Total Sample (M ± SD)	Men (M ± SD)	Women (M ± SD)
Whole grain bread (Pane integrale)	0.09 ± 0.34	0.08 ± 0.32	0.11 ± 0.37
Semolina type and other bread (Pane bianco)	1.28 ± 0.66	1.31 ± 0.65	1.24 ± 0.67
Pasta, rice, polenta (Pasta, riso, polenta)	0.52 ± 0.26	0.53 ± 0.26	0.50 ± 0.25
Desserts (Torte, crostate)	0.17 ± 0.17	0.16 ± 0.16	0.20 ± 0.18
Low-fat meat (Carne magra)	0.34 ± 0.11	0.35 ± 0.11	0.34 ± 0.11
Fatty meat (Carne di maiale)	0.12 ± 0.14	0.13 ± 0.14	0.09 ± 0.14
Poultry, rabbit (Pollame e coniglio)	0.25 ± 0.15	0.25 ± 0.15	0.25 ± 0.15
Cotechino, zampone (Cotechino, zampone)	0.005 ± 0.033	0.005 ± 0.03	0.006 ± 0.04
Cured meat, sausages (Salumi, salsiccia)	0.13 ± 0.15	0.14 ± 0.15	0.11 ± 0.15
Lean ham (Prosciutto magro)	0.13 ± 0.14	0.14 ± 0.15	0.12 ± 0.14
Fatty ham, ham (Prosciutto grasso o cotto)	0.13 ± 0.14	0.14 ± 0.14	0.13 ± 0.14
Bacon, cheek lard (Pancetta e guanciale)	0.04 ± 0.07	0.04 ± 0.08	0.03 ± 0.07
Meat sauce (Sughi di carne, ragù)	0.32 ± 0.12	0.33 ± 0.12	0.32 ± 0.11
Fish (Pesce)	0.23 ± 0.15	0.23 ± 0.15	0.23 ± 0.15
Seafoods (Frutti di mare)	0.07 ± 0.10	0.07 ± 0.12	0.06 ± 0.08
Whole milk (Latte intero)	0.25 ± 0.45	0.25 ± 0.45	0.26 ± 0.45
Skimmed milk, low fat milk (Latte scremato o parzialmente scremato)	0.26 ± 0.44	0.24 ± 0.43	0.28 ± 0.46
Sheep ricotta, fiordilatte, mozzarella, smoked provola (Ricotta di pecora, fiordilatte, mozzarella, provola affumicata)	0.29 ± 0.18	0.27 ± 0.19	0.31 ± 0.16
Cow ricotta (Ricotta di vacca)	0.15 ± 0.15	0.13 ± 0.14	0.17 ± 0.15
Other cheeses (Altri formaggi)	0.27 ± 0.18	0.27 ± 0.19	0.27 ± 0.18
Eggs (Uova)	0.20 ± 0.16	0.19 ± 0.16	0.20 ± 0.16
Fried foods (Alimenti fritti)	0.18 ± 0.15	0.19 ± 0.15	0.18 ± 0.15
Chocolate (Cioccolata)	0.07 ± 0.12	0.06 ± 0.13	0.07 ± 0.12
Croissant, buns, pizzas (cornetti, maritozzi, pizza)	0.04 ± 0.11	0.04 ± 0.13	0.03 ± 0.10
Typical local rotisserie (Supplì, calzone, arancini)	0.08 ± 0.09	0.08 ± 0.10	0.08 ± 0.09
Sandwiches (Tramezzini)	0.01 ± 0.06	0.02 ± 0.07	0.01 ± 0.04
Legumes: peas, beans, lentils, fava beans, chickpeas (Legumi: piselli, fagioli, lenticchie, fave, ceci)	0.32 ± 0.11	0.32 ± 0.11	0.31 ± 0.12
Raw vegetables, Cooked vegetables (Verdure crude e cotte)	0.41 ± 0.23	0.39 ± 0.23	0.43 ± 0.24
Fresh fruit (Frutta fresca)	1.81 ± 0.50	1.76 ± 0.55	1.88 ± 0.40

**Table 3:** Cox regression model of mortality for foods (per one frequency per day) in the questionnaire, adjusted for covariates *.

Foods in Questionnaire	HR of Mortality for one Frequency Per Day, Adjusted for Covariates (95% CI)
Whole grain bread	1.10 (0.93 to 1.30)
Semolina type band other bread	0.92 (0.83 to 1.02)
Pasta, rice, polenta	0.92 (0.71 to 1.18)
Desserts	0.89 (0.58 to 1.37)
Low-fat meat	0.86 (0.48 to 1.53)
Fatty meat	**0.60 (0.37 to 0.99)**
Poultry, rabbit	1.02 (0.67 to 1.54)
Cotechino, zampone	0.16 (0.01 to 2.07)
Cured meat, sausages	0.78 (0.47 to 1.28)
Lean ham	0.82 (0.50 to 1.35)
Fatty ham, ham	**0.59 (0.35 to 0.99)**
Bacon, cheek lard	0.72 (0.25 to 2.09)
Meat sauce	1.55 (0.85 to 2.82)
Fish	0.80 (0.53 to 1.22)
Seafood	0.63 (0.33 to 1.22)
Whole milk	1.07 (0.93 to 1.23)
Skimmed milk, low-fat milk	1.00 (0.87 to 1.15)
Sheep ricotta, fiordilatte, mozzarella, smoked provola	**1.36 (0.99 to 1.86)**
Cow ricotta	1.45 (0.94 to 2.24)
Other cheeses	1.05 (0.73 to 1.52)
Eggs	**0.63 (0.42 to 0.95)**
Fried foods	1.11 (0.72 to 1.71)
Chocolate	1.05 (0.56 to 1.97)
Croissant, buns, pizzas	0.55 (0.24 to 1.27)
Typical local rotisserie	0.89 (0.42 to 1.87)
Sandwiches	0.79 (0.16 to 3.75)
Legumes: peas, beans, lentils, fava beans, chickpeas	1.17 (0.68 to 2.01)
Raw vegetables, cooked vegetables	1.06 (0.80 to 1.41)
Fresh fruit	1.00 (0.88 to 1.15)

Statistical significance is represented in bold. * Model corrected for sex (male, female), age (yrs), BMI (unit), education (low, medium, high), smoking (yes, no), comorbidity (≥2 vs. <2), wine (≥1 L/day vs. <1 L/day), olive oil, (extensive use vs. non-extensive use).

**Table 4:** Cox regression model of mortality for food groups, selected foods, and a posteriori and priori food patterns, adjusted for covariates *.

Variables	HR ^*^ with Olive oil as Adjustment (95% CI)
**Food groups (sum of frequencies)**	(per one frequency per day, not mutually adjusted)
Legumes and vegetables (Legumi e vegetali)	1.07 (0.85 to 1.36)
Fresh cheese (Ricotta di pecora e di vacca, fiordilatte, mozzarella, provola affumicata)	**1.29 (1.03 to 1.63)**
Red meat (Carne rossa: carne magra e grassa)	0.89 (0.66 to 1.19)
Processed meat (Carni processate: salumi, salsicce, pancetta, guanciale, cotechino, zampone, prosciutto magro e grasso o cotto)	0.85 (0.70 to 1.03)
Fish and seafood (pesce e frutti di mare)	0.78 (0.57 to 1.07)
Sweets (Torte, crostate, cioccolata, prodotti confezionati)	0.89 (0.58 to 1.37)
**First 5 foods after backward selection**	(per one frequency per day, mutually adjusted)
Fatty meat (Carne grassa)	0.64 (0.39 to 1.06)
Fatty ham, baked ham (Carne grassa e cotta)	**0.59 (0.35 to 0.99)**
Meat sauce (Sughi di carne, ragù)	1.76 (0.97 to 3.20)
Sheep ricotta, fiordilatte, mozzarella, smoked provola (Ricotta di pecora, fiordilatte, mozzarella, provola affumicata)	**1.41 (1.04 to 1.92)**
Eggs (Uova)	**0.62 (0.41 to 0.94)**
**First 5 RSF foods with highest importance**	(per one frequency per day, mutually adjusted)
Semolina type and other bread (Pane bianco)	0.94 (0.84 to 1.04)
Pasta, rice, polenta (Pasta, riso, polenta)	0.93 (0.73 to 1.20)
Desserts (Dolci, crostate, etc.)	0.96 (0.62 to 1.50)
Fatty meat (Carne grassa)	0.63 (0.38 to 1.04)
Poultry, rabbit (Pollame, coniglio)	0.99 (0.65 to 1.50)
**Components of PCA**	(per pca-score, not mutually adjusted)
Energy-Rich Foods Pattern	0.96 (0.92 to 1.01)
Farmhouse Diet Pattern	**1.05 (1.00 to 1.10)**
Sweets Pattern	1.01 (0.95 to 1.08)
Winter Pattern	0.97 (0.92 to 1.02)
Elderly Pattern	1.01 (0.96 to 1.07)
**A priori indices**	(per index point, not mutually adjusted, excluding olive oil as covariate)
DASH	1.03 (0.97 to 1.10)
MedDiet score	0.98 (0.97 to 1.00)
MIND	0.96 (0.92 to 1.00)
Italian MedDiet index	0.95 (0.90 to 1.00)

Statistical significance is represented in bold. * Model corrected for sex (male, female), age (yrs), BMI (unit), education (low, medium, high), smoking (yes, no), comorbidity (≥2 vs. <2), wine (≥1 L/day vs. <1 L/day), olive oil, (extensive use vs. non-extensive use).

**Table 5:** Multiple Cox regression model of mortality, model adjusted *.

Parameters	Total Mortality	Cancer Mortality	Cardiovascular and Stroke Mortality
	HR (95% CI)	HR (95% CI)	HR (95% CI)
Components PCA ^§^			
Farmhouse Diet Pattern	**1.05 (1.00 to 1.10)**	1.03 (0.93 to 1.13)	0.98 (0.88 to 1.10)
Foods ^§^			
Fatty meat	0.89 (0.76 to 1.05)	0.97 (0.71 to 1.33)	0.79 (0.55 to 1.15)
Dairy products			
Sheep and cow ricotta, fiordilatte, mozzarella, smoked provola	1.29 (1.03 to 1.63)	1.42 (0.90 to 2.22)	0.96 (0.57 to 1.61)
Eggs	**0.86 (0.76 to 0.99)**	0.87 (0.66 to 1.13)	**0.73 (0.55 to 0.98)**
Fatty ham, baked ham	**0.59 (0.35 to 0.99)**	0.90 (0.35 to 2.32)	0.44 (0.14 to 1.45)

Statistical significance is represented in bold. Abbreviation: HR,Hazard Ratio; se(HR), standard error of HR; BMI, Body Mass Index; PCA, Principal Component Analysis. * Model corrected for: Sex (Female), Age (years), BMI, Education (Low, Medium, High), Smoking (yes), Comorbidity (≥ 2), Wine (≥ 1lt/day), and Olive oil, (No use or little) vs (Much use). § Parameters individually insert in the model.
